# Commissioning for health and well-being: an introduction

**Published:** 2012-08-10

**Authors:** Marie-Jeanne Aarts

**Affiliations:** Department of Health Services Research, CAPHRI School of Public Health and Primary Care, Maastricht University, P.O. Box 616, 6200 MD Maastricht, The Netherlands E-mail: mj.aarts@maastrichtuniversity.nl

Both in health and social care, commissioning is a term that has been used increasingly as an answer to many of today’s challenges in these policy fields. However, it is not always clear what commissioning for health and well-being exactly encompasses and how it can be put into action to achieve the best results and outcomes. This book gives an overview about what commissioning for health and well-being is, the main methods involved, and the main future challenges for practice as well as research.

The book is written by 17 authors all based or associated with the University of Birmingham. After an introductory chapter by the editor, which outlines the aims and scope of the book and some key definitions of commissioning for health and well-being, the first part of the book focuses on the commissioning cycle. The second part of the book addresses some key underlying themes, whereas the third part of the book describes the overall conclusions and next steps for commissioning in health and social care. In total this book contains twelve chapters, which will be addressed in more detail below. Each chapter start with a short and general summary consisting of bullet points and ends with a concluding paragraph, suggestions for further reading, useful websites and some reflective exercises for the reader.

The first chapter chronicles the development from commissioning to strategic commissioning in the UK. Based on some practical examples such as ‘Every child matters’ and ‘Work and pensions’, different forms of the commissioning cycle are presented. However, no general definition of what commissioning actually is, can be given, as this differs across government departments. The second chapter explores the importance and nature of needs assessment and highlights the difference between supply, demand and need. The chapter further describes the three main ways to undertake health care needs assessment: epidemiological, corporate and comparative needs assessment. Chapter 3 deals with the key features of resource allocation and priority setting in the UK, including economic approaches such as health technology assessment. Furthermore, Matland’s ambiguity-conflict model is discussed in relation to the challenges that arise when seeking to implement policy decisions. The chapter concludes however, that there is no ‘magic bullet’ or ‘one-size-fits-all’ solution for priority setting on the local level.

The fourth chapter, which is titled “Procurement and market management”, addresses how organizations try to get best value for money, and several difficulties that can arise during this process, such as opportunism and difficulties within negotiation. Chapter 5 uses the Maslin Multi-Dimensional Matrix (MMDM) to illustrate how to decide on decommissioning services, an often underexplored theme, but increasingly important within the light of recent economic trends. The MMDM can assist policy-makers to take into account different dimensions in making decisions on decommissioning services and the chapter gives some practical UK examples of how to apply the MMDM.

In contrast to the previous chapter, chapter 6 focuses on commissioning for services resilience and elaborates on the question why some providers fail to adapt to changing environments while others are more successful. Again, the MMDM approach can be used to provide valuable insights. Like in the previous chapters, some practical examples from the UK are extensively discussed to illustrate the main points addressed in this chapter. Although commissioning in health and social care has often been mentioned to increase quality of services, in fact there is no consensus on which outcomes should be monitored as indicators of quality. In chapter 7, outcome-based commissioning cycle is discussed and evidence from research addressing outcome-focused commission is summarized. The main conclusion of these studies is that high quality commissioning should be about people instead of services. With an increased demand on health and social care combined with cuts in governmental budgets to be expected over the next few years, commissioning will also have to rely on people’s own responsibilities in getting good quality services.

Chapter 8 explores the economics of commissioning by discussing the different roles of the commissioner in a market system. From the discussion in this chapter it emerges that commissioning services may be demanded in a private market situation in order to assist the customer while dealing with information asymmetry, by providing expertise, diagnosis and the monitoring of quality, or in conjunction with insurance. The chapter also addresses market failure, in case of monopoly, public goods problems and equity considerations. The ninth chapter starts with the observation that patients and the public have become increasingly involved in the planning, development and improvement of health and social services, and that this patient and public involvement (PPI) can take different forms. Next to the traditional ladder of participation as presented already in 1969 by Arnstein, other typologies of public involvement are discussed in this chapter.

Because health and social care services often overlap, chapter 10 focuses on joint commissioning in these two policy fields. The authors state that “despite all the rhetoric about what joint commissioning *might* produce, there is surprisingly little evidence to support this”. The chapter discusses difficulties in evaluating joint commissioning. Chapter 11 zooms in on personalization within the field of commissioning, in other words “starting with the person rather than the service”. The most prominent example of personalization is perhaps when users hold full responsibility for their own budget to be allocated to health or social care services. Although highly flexible for users, the lack of block contracts however poses challenges for providers of services. The chapter ends with an exploration of future scenarios of personalized commissioning. Chapter 12 finally wraps up with a summary of all the themes addressed in this book.

Overall, this book is worth reading for professionals, policy-makers and researchers in the field of integrated care that have to deal with commissioning in, but because of the strong focus on the UK setting, this book is not well suited for international readers. However, the book gives a comprehensive overview of different themes related to commissioning and the combination of theoretical viewpoint and practical examples, which makes it interesting to read for UK readers.

